# Evaluation of self-swabbing coupled with a telephone health helpline as an adjunct tool for surveillance of influenza viruses in Ontario

**DOI:** 10.1186/s12889-016-3674-9

**Published:** 2016-09-27

**Authors:** D. McGolrick, P. Belanger, H. Richardson, K. Moore, A. Maier, A. Majury

**Affiliations:** 1 Queen’s University, Kingston, Ontario Canada; 2 Kingston, Frontenac, and Lennox & Addington Public Health, Kingston, Ontario Canada; 3 Public Health Ontario, Kingston, Ontario Canada

**Keywords:** Surveillance, Self-swabbing, Influenza viruses, Respiratory viruses, Community-based, Ontario

## Abstract

**Background:**

Calls to a telephone health helpline (THHL) have been previously evaluated for the ability to monitor specific syndromes, such as fever and influenza-like-illness or gastrointestinal illness. This method of surveillance has been shown to be highly correlated with traditional surveillance methods, and to have potential for early detection of community-based illness. Self-sampling, or having a person take his/her own nasal swab, has also proven successful as a useful method for obtaining a specimen, which may be used for respiratory virus detection.

**Methods:**

This study describes a self-swabbing surveillance system mediated by a nurse-led THHL in Ontario whereby syndromic surveillance concepts are used to recruit and monitor participants with influenza-like illness. Once recruited, participants collect a nasal specimen obtained by self-swabbing and submit for testing and laboratory confirmation. Enumeration of weekly case counts was used to evaluate the timeliness of the self-swabbing surveillance system through comparison to other respiratory virus and influenza surveillance systems in Ontario. The operational efficiency of the system was also evaluated.

**Results:**

The mean and median number of days between the day that a participant called the THHL, to the day a package was received at the laboratory for testing were approximately 10.4 and 8.6 days, respectively. The time between self-swab collection and package reception was 4.9 days on average, with a median of 4 days. The self-swabbing surveillance system adequately captured the 2014 influenza B season in a timely manner when compared to other Ontario-based sources of influenza surveillance data from the same year; however, the emergence of influenza B was not detected any earlier than with these other surveillance systems. Influenza A surveillance was also evaluated. Using the THHL self-swabbing system, a peak in the number of cases for influenza A was observed approximately one week after or during the same week as that reported by the other surveillance systems.

**Conclusion:**

This one-year pilot study suggests that the THHL self-swabbing surveillance system has significant potential as an adjunct tool for the surveillance of influenza viruses in Ontario. Recommendations for improving system efficacy are discussed.

**Electronic supplementary material:**

The online version of this article (doi:10.1186/s12889-016-3674-9) contains supplementary material, which is available to authorized users.

## Background

Influenza viruses circulate worldwide and affect people of all ages causing significant morbidity and mortality annually. Influenza is believed to spread primarily through direct person-to-person interactions by way of large droplets (>5 μm) that are generated when a person coughs, sneezes, or talks. The large respiratory droplets that are released into the air can then be inhaled or ingested. Influenza viruses have also been noted to spread indirectly through contaminated hands or shared surfaces [1]. Infection with influenza virus leads to an acute respiratory disease characterized by the sudden onset of high fever, coryza, cough, headache, prostration, malaise, and inflammation of the upper respiratory tract [2, 3]. While the majority of those who are infected with the virus only experience mild symptoms, influenza can lead to severe infection in others.

It is estimated that between 10 and 20 % of Canadians become infected with influenza each year. In the temperate climates of the Northern Hemisphere, including Canada, outbreaks generally occur annually between December and March. These outbreaks or “epidemics” usually last 8–10 weeks and include one or two “waves” or peaks of influenza cases with the first wave often the result of influenza A viruses succeeded by a second wave of influenza B viruses [1, 4].

In Canada, during the 2014–2015 influenza season, which includes September through August, 951 hospitalizations and eight deaths were reported among the pediatric population (≤19 years), and 6991 hospitalizations and 597 deaths were reported among the adult population (≥20 years) [5]. Presumably these numbers only represent a subset of all influenza-associated hospitalizations and deaths, according to approximations that have been made with national datasets, suggesting the true number of influenza-related hospitalizations in Canada approximates 12,200 per year, and the number of deaths attributable to the disease is closer to 3500 per year [6, 7]. Given the impact of influenza on the health care system and the health of Canadians, it is important to monitor influenza viruses with the intention of preventing a widespread outbreak, especially within the community where epidemics begin and from which they spread. In addition, monitoring communicable diseases provides valuable information that is useful for multiple reasons, including: vaccine development and evaluation, allocation of health care associated resources, preparing health care systems for a surge of cases, tracking disease spread, informing clinicians, and preventing and containing outbreaks.

### Linking telephone health helpline data to laboratory data

National Health Service (NHS) Direct is a national THHL service provided by the NHS of the United Kingdom that was used to relay health information to callers from England and Wales, providing those in need with advice regarding the appropriate health care services that should be sought [8]. NHS Direct was explored as a syndromic surveillance tool between 2001 and 2003 with an initial focus on detecting a chemical or biologic attack by monitoring a range of algorithms related to symptoms that would relate to such an attack [8, 9]. This method was successful for detecting elevated levels of symptom-specific activity; however, the sole use of symptomatic data to detect an outbreak highlighted certain limitations, such as the lack of a patient sample for laboratory confirmation of the agent of interest. This limitation was later addressed by studies that demonstrated the feasibility of community-based virological self-sampling, or self-swabbing, in conjunction with NHS Direct [10]. Self-swabbing was both acceptable to participants and feasible with regards to providing appropriate samples for molecular testing based on both available participant data and the test results from the returned swabs [10]. A follow-up, opportunistic study was carried out in England during May and June of 2009, using community self-sampling of callers to NHS Direct. The purpose of this study was to evaluate whether the onset of community transmission of pandemic influenza A (A(H1N1)pdm09) was detected during its earliest phase by comparing data from self-sampling to data routinely collected by the Health Protection Agency (HPA) [11]. This study highlighted that the data collected via self-sampling was similar to that collected by the HPA in that they both provided a reliable indication of the extent local community transmission was occurring. Additionally, transmission of seasonal influenza A (H3) and B viruses early in the summer was also captured, which is a trend that is not commonly observed.

### Self-sampling for the detection of respiratory viruses

To support the validity of virological evidence derived from self-sampling, a series of studies have been conducted to evaluate nasal swabs collected through self-sampling in comparison to clinically derived samples. In 2011, the feasibility of using nasal self-sampling for population-based surveillance of respiratory virus infections in the adult working population was explored through a cohort study performed in Eskilstuna, Sweden [12]. Participants were asked to collect a sample, which was then submitted and tested for 14 viruses, upon the onset of one or more of the following symptoms: fever (>38 °C), upper respiratory tract infection, and gastroenteritis. A total of 876 nasal swabs (47.5 %) contained at least one virus, and the proportion of positive tests for specific viruses was shown to be similar to what was collected clinically.

In an England-based study during the 2009 influenza pandemic, self-sampling was implemented to support community-based virological surveillance of influenza between May 2009 and March 2010 [13]. Participants aged 16 or older were recruited for the self-sampling scheme if they had used NHS Direct and/or the website interface for cold/influenza symptoms, and those who agreed to participate submitted a self-obtained nasal swab that was analysed by real-time RT-PCR for influenza A(H1N1)pdm09, influenza A(H1N1), A(H3N2), and influenza B. The results from participant-derived swabs were compared to swabs taken by a clinician, which demonstrated that there was no significant difference in cycle threshold (Ct) values between self-sampling and clinician-led sampling [13]. The equivalence of self- and staff-collected nasal swabs was also studied among employees at the Helmholtz Centre for Infection Research in Germany, which reported that β-actin DNA levels and the percentage of swabs from which a pathogen was detected were both slightly higher in self-collected specimens when compared to staff-collected specimens [14]. Both of these studies support the use of self-sampling as an alternative method for obtaining surveillance data about respiratory viruses.

Lastly, e-mail-based active syndromic surveillance with nasal self-swabbing has also been evaluated for the detection of viral respiratory pathogens [15]. Self-swabbing was deemed highly feasible in terms of “acceptance, satisfaction, compliance, and timeliness” and that delayed testing as a result of the delay between symptom onset, self-swabbing, and specimen arrival at the laboratory did not influence virus detection rates.

### Aim of this study

The purpose of this study was to explore the use of Ontario’s THHL, combined with self-swabbing, for the early detection of circulating influenza viruses and to evaluate its use as a community-based surveillance tool in Ontario. Currently, in Ontario, community surveillance for influenza viruses is limited at best as it requires patients to present for health care services to collect data, thus this system allows for the surveillance of a previously un-surveyed population. Earlier studies have shown that the number of calls to telephone health helplines for specific syndromes such as fever and influenza-like-illness (ILI) or gastrointestinal illness are highly correlated with traditional surveillance methods and may detect outbreaks earlier [16, 17]. Moreover, self-swabbing, or having the person take his or her own nasal swab, has proven successful as a useful method for obtaining a specimen for respiratory virus detection [12, 18]. The self-swabbing surveillance system was evaluated as an early detection surveillance system in comparison to the Public Health Agency of Canada’s (PHAC’s) FluWatch, Public Health Ontario (PHO) laboratory data, and Ontario’s Acute Care Enhanced Surveillance (ACES) system by comparing the timeliness of each system in terms of the ability to detect influenza A or influenza B viruses at the earliest possible time. This study also evaluated both the feasibility and efficacy of the self-swabbing surveillance system as a surveillance tool through assessment of system functionality in terms of timeliness (early detection and reporting delay).

## Methods

### Recruitment

Using Ontario’s THHL, callers were recruited to participate in the study provided they met the categories of “referral” (contact a family physician within 72 h) or “self-care”. For reference, callers are triaged into five categories: priority (call 911 immediately), emergency (see physician within hours), urgent (contact family physician within 24 h), and the two used for this study. Further, all participants had to be at least two years of age, and experiencing at least one or more of fever, cough, coryza, or sore throat. Recruitment began on October 1, 2013 and continued for one full year until September 30, 2014. At the start of recruitment in October 2013, 30 nurses were involved in the recruitment process. On three subsequent dates, the total number of nurses recuriting participants was increased: five nurses were added during the week of December 29, 2013, for a total of 35 nurses; 85 were added on January 23, 2014, for a total of 120 nurses; and another 32 were added on March 5, 2014, for a final total of 152 nurses participating for the remainder of the recruitment phase of the study. The number of nurses participating in the study was outside of the control of the investigator, and the initial low numbers were consequent to the THHL provider underestimating the need.

### Transfer of data and specimen testing

Upon agreeing to participate, contact information was provided to THHL personnel, which was transferred daily via a secure portal to the research team. A “self-swabbing package” was prepared and shipped out the next business day to the address on file for the participant. Each self-swabbing package contained the following: a flocked swab (Copan Diagnostics Inc., Murrieta, CA), two pre-printed labels with unique study number, a SAFTPAK STP-700 set (one leak proof polybag and one Tyvek® envelope, SAF-T-PAK Inc., Edmonton, AB), a letter of information about the study, a set of instructions, a questionnaire, and a consent form. Additionally, an assent form was included if the participant was under the age of 16. Briefly, the instructions outlined the purpose of the study, directions for completing the required documentation, and how to return the self-swabbing package. It was requested that participants return the nasal swab, questionnaire, and consent (or assent) forms using the enclosed prepaid packaging. Packages that were returned without a completed consent (or assent) form were destroyed and recorded in the database as “returned without consent.”

Upon receipt of the self-swabbing packages by the laboratory, the nasal swabs were stored at 4 °C until tested. Note that participant instructions also recommended storage of swabs at 4 °C following sample collection and prior to return to the laboratory, although this was not controlled for in this study. Nucleic acid extraction was performed using the Nuclisens® EasyMag® (Bioméreiux Inc.) in accordance with the instructions provided by the manufacturer (Nuclisens® EasyMag® User Manual, version 2.0, ref.280163) producing a 25 μL eluate. Specimens were then tested for influenza A, influenza B, and GAPDH (as a control), by RT-PCR using the Viia™7 Real-Time PCR System (Applied Biosystems).

### Timeline

**Fig. 1:**
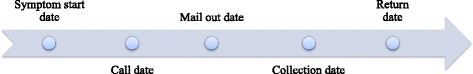
Chronological overview of the timeline used to assess the operation of the self-swabbing study

The “symptom start date,” “call date,” “mail out date,” “collection date,” and “return date” represent points in time throughout the operation of the self-swabbing surveillance study (Fig. 1) and the difference in the number of days between each of the time points was calculated. As the questionnaire database is dependent on participants who returned a self-swabbing package with consent, the symptom start date and collection date were not available for those who did not return with consent. As a result, the difference in the number of days for certain time points returned a value of less than zero and were therefore excluded for analyses. Basic statistics for each of the time points were calculated, which included: number of observations included, mean number of days (and standard deviation), median number of days, and minimum and maximum number of days. The results derived for the difference in time between time points evaluate the efficiency of the self-swabbing surveillance system by providing insight into when delays occurred in the process, how long it took participants to go through the steps of the surveillance system, and how rapidly the self-swab sample could be returned to the laboratory to be tested.

### Time series analyses

In order to assess whether the self-swabbing study was capable of detecting circulating respiratory viruses in Ontario earlier than comparable systems within the province, the surveillance data collected for this study was compared to the equivalent data provided by FluWatch, PHO laboratory data, and ACES. A “case” for the self-swabbing study, FluWatch, and PHO laboratory data was defined as a positive test for the virus of interest. Two datasets were used for the self-swabbing study, one with the raw number of cases by week and another with an adjusted number of cases by week accounting for the increase in the number of nurses that were recruiting participants at that time. These two datasets will subsequently be referred to as the “self-swabbing dataset” and “self-swabbing adjusted dataset”, respectively. The adjusted dataset was calculated using equation 1 to determine the estimated number of participants that would have been recruited had 152 nurses been involved throughout the entire study.

1 $$ adjusted\  number\  of\  participants=\frac{152\kern0.5em \left( raw\  number\  of\  participants\right)}{x} $$

where x = number of nurses recruiting during the week that the respective participants were recruited.

Two datasets were also used for ACES and therefore two “case” definitions were used: the first was defined as a visit to an acute care hospital for a respiratory syndrome, and the second was a visit to an acute care hospital for a fever and/or ILI syndrome. The data from each system was sorted to provide a weekly count of cases; the reporting weeks used are defined in Additional file 1. Based on the data available for this study, a peak comparison method was deemed most appropriate for the time series analyses. In brief, the peak comparison method is a comparison of the time at which a peak of cases or local maximum was observed [19]. The week at which the peak was observed was used to compare periods of circulation of respiratory viruses as detected using the self-swabbing study data, FluWatch, PHO laboratory data, and ACES.

## Results

### Timeline assessment

The manner by which the self-swabbing surveillance system operates involves various time points that have the potential to impact the time it takes to successfully transition through the system. To provide a more comprehensive description of the system’s ability to detect circulating respiratory viruses in a timely manner, the efficiency of the self-swabbing surveillance system was analyzed based on time measurements between the various time points in the process as illustrated in Fig. 1. The mean number of days between the ‘call date’ and ‘return date’ was 10.4 days and the median was 8.6 days. The corresponding minimum and maximum number of days for this interval was 2.2 and 86.6 days. For a more accurate description of the turnaround time, the time between the ‘mail out date’ and ‘return date’ was calculated, which had a slightly lower mean and median of 9.5 and 8.0 days, respectively. The minimum and maximum number of days for this interval was the same as the time between the call date and package return date.

On average, participants waited 4.4 days between the onset of symptoms (‘symptom start date’) to their call to the THHL (‘call date’). The median for this interval was slightly lower at 3.0 days, and the minimum and maximum number of days was 0 and 55; however, the majority (94.9 %) of participants for which data is available called less than 2 weeks after symptom onset. Furthermore, more than half of participants (57.4 %) called within 3 days of symptom onset. After participants called the THHL, it took an average of 6.1 days to collect the nasal swab, or a median of 5.0 days. The minimum and maximum days between call and swab collection were 2.0 and 62.0 days. Lastly, the mean number of days between swab collection (‘collection date’) and returning a package to the testing laboratory (‘return date’) was just under 5.0 days (4.9 days) or a median of 4.0 days. The fastest time between swab collection and returning a specimen was one day and the maximum was 52.0 days. It is important to note that the maximum value is an outlier and does not reflect the distribution of this time interval. Approximately 90 % of the specimens received at the laboratory with a swab collection date reported were received within 8.0 days, and 25 % were received within 3.0 or less days.

### Participant specimen results

A total of 664 out of 666 specimens received with consent were successfully tested over the course of the year. Eighty-seven specimens tested positive for influenza (27 influenza A and 60 influenza B) corresponding to a 13.1 % positivity rate. In comparison, FluWatch had a very similar overall positivity rate of 13.7 % in Ontario for the same time period. Moreover, when samples from peak influenza season only were evaluated (December 2013 to March 2014), an 18.6 % positivity rate was observed (60 positives: 27 influenza A and 33 influenza B). Again, this highly resembled the Ontario data derived from FluWatch, which had an overall positivity rate during peak influenza season of 18.4 %.

### Peak comparison

**Fig. 2:**
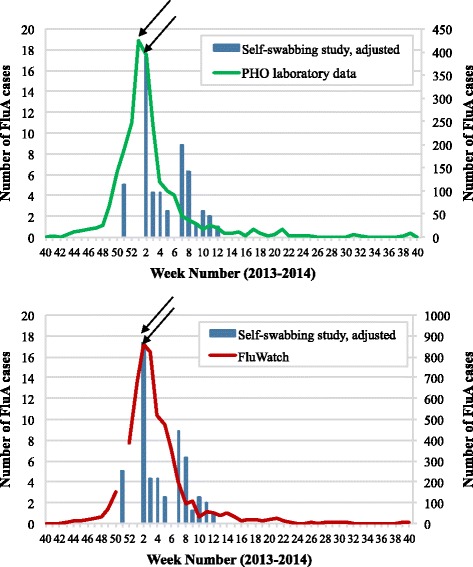
The weekly case counts for influenza A (FluA) viruses detected between October 1, 2013 and September 30, 2014 by: the self-swabbing study (adjusted dataset) using the date a specimen was tested, and PHO laboratory data (*top*) and FluWatch (*bottom*) data. Arrows were used to indicate when the peak number of cases occurred for each dataset. PHO laboratory data and FluWatch data were plotted on the secondary axis

First, the weekly case count for influenza A viruses was examined. Both the self-swabbing dataset and self-swabbing adjusted dataset were used to highlight the difference between the timing of the curves observed for the two datasets, with the maximum number of cases having occurred five weeks earlier at week 2 using the adjusted dataset. In addition, an artificial smaller peak of influenza A cases during week 2 was observed using the raw dataset, which can be attributed to the increase in recruiting nurses as the global maximum number of cases using this dataset was during week 7. In comparison to the weekly case count for influenza A viruses reported by the PHO laboratory data that peaked during week 1, the peak for the self-swabbing adjusted dataset was delayed by one week (Fig. 2, top). The self-swabbing study data was also compared to FluWatch data, which reached a maximum weekly number of cases at the same time as the adjusted number of cases determined by the self-swabbing study during week 2 (Fig. 2, bottom).

**Fig. 3:**
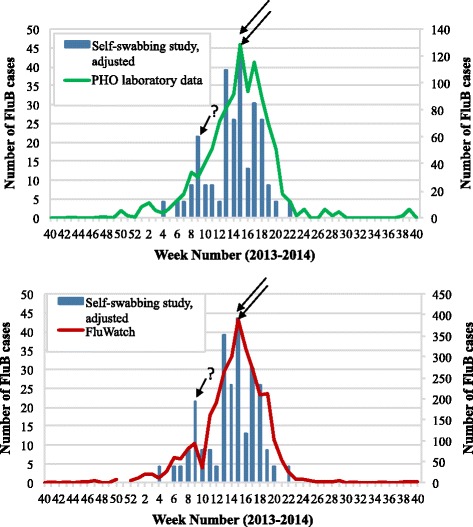
The weekly case counts for influenza B (FluB) viruses detected between October 1, 2013 and September 30, 2014 by: the self-swabbing study (adjusted dataset) using the date a specimen was tested, and PHO laboratory data (*top*) and FluWatch (*bottom*) data. Arrows were used to indicate when the peak number of cases occurred for each dataset. PHO laboratory data and FluWatch data were plotted on the secondary axis

Next, the weekly case count for influenza B viruses was examined. Only the self-swabbing adjusted dataset was included as the difference between the raw and adjusted numbers had far less of an impact on the seasonal curves observed for the influenza B season than the equivalent for influenza A. This is because the change made to the number of recruiting nurses was smaller during the influenza B season, for example, there were 120 nurses participating as of week 4 and 152 by week 10. The number of influenza B cases was compared to laboratory derived data and FluWatch data, which both had a maximum number of cases occur in week 15 with 129 and 389 cases, respectively (Fig. 3). The maximum number of influenza B cases was also observed during week 15 with 43 cases according to the adjusted dataset. Interestingly, the self-swabbing study appeared to have a smaller peak in the number of cases prior to the global maximum, which occurred at week 9 with 22 cases or six weeks earlier than PHO laboratory data. The earlier rise in influenza cases was also recorded by FluWatch data during week 9.

**Fig. 4:**
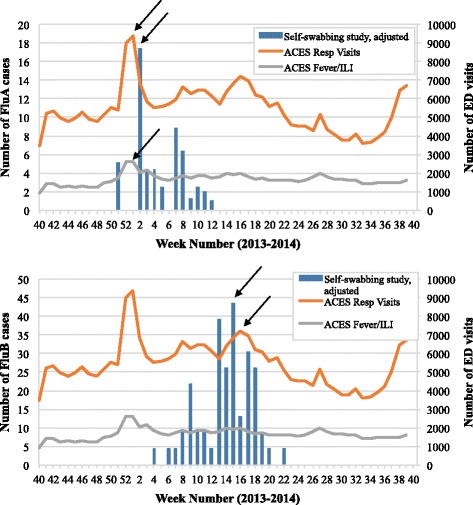
The weekly case counts for influenza A viruses (FluA, *top*) and influenza B viruses (FluB, *bottom*) as detected by the self-swabbing study (adjusted dataset) using the date a specimen was tested, compared to the weekly number of respiratory (resp) visits and fever and ILI (fever/ILI) visits monitored by ACES from October 1, 2013 to September 30, 2014. Arrows were used to indicate when the peak number of cases occurred for each dataset. ACES data was plotted on the right axis

Lastly, the self-swabbing study was compared to two different measures derived from ACES data: the number of respiratory visits, and the number of fever and ILI visits to emergency departments (EDs) in Ontario. The ACES system uses respiratory, and fever and ILI visits as an indicator for increased respiratory disease activity. In addition to the PHO laboratory data and FluWatch data, these indicators were also used for the evaluation of the self-swabbing system’s ability to monitor weekly case counts of influenza A and influenza B viruses in a timely manner. Only the self-swabbing adjusted datasets were included in Fig. 4 as the increased number of nurses was previously addressed . The number of respiratory visits reached a maximum during the same week as the number of fever and ILI visits in week 1, with 9365 visits and 2622 visits, respectively. Both peaks occurred 1 week earlier than the peak in cases of influenza A virus detected by the self-swabbing adjusted dataset, and 6 weeks earlier than the data from the self-swabbing study using the raw number of cases. ACES detected only one distinct peak in the number of visits over the course of the 2013 to 2014 influenza season, which occurred in week 1; however, a smaller, less defined increase in respiratory visits was also observed during week 16 with 7174 visits. Nonetheless, the weekly case count for influenza B virus that was determined by the self-swabbing study occurred 1 week earlier in week 15.

## Discussion

In summary, the self-swabbing study was able to detect the seasonality of influenza A viruses at approximately the same time as FluWatch (week 2) and 1 week later than the provincial laboratory based data (week 1) when using the self-swabbing adjusted dataset; however, it did not capture the same trends any earlier than methods currently used in practice. With respect to influenza B viruses, the self-swabbing study was capable of capturing the seasonal trend at the same time as other systems currently used in practice. While the maximum number of cases determined by the self-swabbing study did not occur any earlier than other sources, a different trend involving a smaller peak earlier in the season was observed, which was not apparent based on the provincial laboratory data. These preliminary results show that the self-swabbing study may have potential for enhancing current surveillance practices. Additionally, the maximum weekly case count for influenza B virus determined by the self-swabbing study occurred 1 week earlier during week 15, in comparison to ACES data. This, in combination with the detection of a different trend, may suggest that the self-swabbing surveillance system was better suited for the detection of circulating influenza B viruses.

The performance of the self-swabbing surveillance system for monitoring influenza A viruses was less than what was anticipated, as the maximum number of cases was observed at a later week than what was observed for the other systems in question. The change in the number of nurses over the course of the study, particularly during the height of influenza A season, was suspected to have had an impact on the weekly counts of influenza that consequently affected the performance of the system; however, this could not be confirmed as information regarding the recruitment rate of eligible callers was not available. When the raw case count was used for analysis, the self-swabbing study was a minimum of 5 weeks later than any other system, but when the numbers were adjusted to account for the increased number of nurses, the peak detected by the self-swabbing study was found to occur at the same time as FluWatch during week 2 and 1 week later than provincial laboratory data and ACES. The lack of recruiting experience among the nurses early in the study versus much later may also have impacted these outcomes, such as evidenced by the more successful monitoring of influenza B virus using both the raw and adjusted datasets for the number of recruiting nurses.

The average number of days from call date to collection date and mail out date to return date was 6.1 and 9.5 days, respectively. It is difficult to control for the amount of time it takes to transfer a specimen, but it is important to note that only one testing site was used for this study. Samples were required to travel from across the entire province of Ontario to this single site as opposed to having multiple, more readily accessible sites available for specimen submissions. As such, it is likely that given the proximity of the testing site to the participant, employing multiple testing sites distributed throughout the province would consequently lead to a significant reduction in the time from sample collection, to shipping, to processing, for these other samples. Another intervention that could be used to reduce the time taken to obtain data for the surveillance system would be to not only include multiple testing sites, but multiple sites for participants to return the self-swabbing packages to. For example, pharmacies or local Public Health Units could facilitate the transportation of packages to testing sites by acting as a pick-up/drop-off location for the self-swabbing packages. By making these adjustments to increase the proximity of the testing site to the participant, this would likely improve the efficacy of self-swabbing as an early detection method for surveillance. The magnitude of such improvements is recommended for future investigation.

While addressing the timeliness of the system, it is also worth considering that the surveillance system under study was not associated with any advertisements or follow-up reminders to prompt participants to return a package. The participant following through with the study was reliant on the participant remembering to act once a package was received. It would be valuable to investigate whether incorporating a follow-up procedure into the study, such as an automated e-mail or phone reminder, would have an impact on improving the response rate and reducing the time it took for participants to return a package.

Self-swabbing also has the potential to provide an opportunity for the integration of public health and primary care, and antimicrobial stewardship. A number of countries, including Canada, have, and continue to, invest in improving the coordination between public health and primary care, as both are working towards a common goal and share healthcare responsibilities, particularly at the community level [20]. The implementation of a self-swabbing surveillance system provides an opportunity for public health authorities to generate diagnostic information that can be relayed to physicians. Further, this diagnostic information will aid in the practice of antimicrobial stewardship, which is an ongoing issue especially as it pertains to the inappropriate prescription of antimicrobials for the treatment of viral infections. A laboratory diagnosis promotes the rational use of antiviral agents and discourages the inappropriate prescription of antibiotics, which could in turn potentially reduce inappropriate treatment of influenza-like illness in up to 50 % of cases [21].

## Conclusions

In conclusion, this one-year pilot study, which explored the use of obtaining specimens viable for testing and collected by self-swabbing through the use of a THHL, has been shown to be a practical method for monitoring influenza viruses in Ontario. Self-swabbing mediated by a THHL also has the potential added benefit of reducing the number of visits to physician’s offices and emergency departments, which would in turn contribute to a reduction of the rate of viral transmission in a community. Furthermore, based on its ability to capture community-based cases in a timely manner, as well as enabling surveillance of the community based population which is currently not surveyed, it would be a beneficial addition the surveillance of influenza viruses in Ontario. Further investigation into the surveillance system described in this study is underway and is anticipated to add to more comprehensive support for future recommendations regarding the implementation of a community-based surveillance practice similar to the self-swabbing surveillance system.

## Additional file

Additional file 1: Reporting weeks for the 2013-2014 surveillance season. (DOCX 15 kb)

## Abbreviations

ACES: Acute Care Enhanced Surveillance
Ct: Cycle threshold
DNA: Deoxyribonucleic acid
ED: Emergency Department
FluA: Influenza A
FluB: Influenza B
HPA: Health Protection Agency
ILI: Influenza-like-illness
NHS: National Health Service
PHAC: Public Health Agency of Canada
PHO: Public Health Ontario
Resp: Respiratory
THHL: Telephone health helpline
