# Supplementary material for: Evaluation of self-swabbing coupled with a telephone health helpline as an adjunct tool for surveillance of influenza viruses in Ontario
Source: BMC Public Health. 2016 Sep 27;16:1017. doi: 10.1186/s12889-016-3674-9 (PMC5039901; doi:10.1186/s12889-016-3674-9)
Supplement: Additional file 1: — Reporting weeks for the 2013-2014 surveillance season. (DOCX 15 kb) [file 12889_2016_3674_MOESM1_ESM.docx]

**Additional file 1.** Reporting weeks for the 2013-2014 surveillance season.

| **Week** | **Start** | **End** |
| --- | --- | --- |
|  |  |  |
| 35 | 25-Aug-13 | 31-Aug-13 |
| 36 | 01-Sep-13 | 07-Sep-13 |
| 37 | 08-Sep-13 | 14-Sep-13 |
| 38 | 15-Sep-13 | 21-Sep-13 |
| 39 | 22-Sep-13 | 28-Sep-13 |
| 40 | 29-Sep-13 | 05-Oct-13 |
| 41 | 06-Oct-13 | 12-Oct-13 |
| 42 | 13-Oct-13 | 19-Oct-13 |
| 43 | 20-Oct-13 | 26-Oct-13 |
| 44 | 27-Oct-13 | 02-Nov-13 |
| 45 | 03-Nov-13 | 09-Nov-13 |
| 46 | 10-Nov-13 | 16-Nov-13 |
| 47 | 17-Nov-13 | 23-Nov-13 |
| 48 | 24-Nov-13 | 30-Nov-13 |
| 49 | 01-Dec-13 | 07-Dec-13 |
| 50 | 08-Dec-13 | 14-Dec-13 |
| 51 | 15-Dec-13 | 21-Dec-13 |
| 52 | 22-Dec-13 | 28-Dec-13 |
| 1 | 29-Dec-13 | 04-Jan-14 |
| 2 | 05-Jan-14 | 11-Jan-14 |
| 3 | 12-Jan-14 | 18-Jan-14 |
| 4 | 19-Jan-14 | 25-Jan-14 |
| 5 | 26-Jan-14 | 01-Feb-14 |
| 6 | 02-Feb-14 | 08-Feb-14 |
| 7 | 09-Feb-14 | 15-Feb-14 |
| 8 | 16-Feb-14 | 22-Feb-14 |
| 9 | 23-Feb-14 | 01-Mar-14 |
| 10 | 02-Mar-14 | 08-Mar-14 |
| 11 | 09-Mar-14 | 15-Mar-14 |
| 12 | 16-Mar-14 | 22-Mar-14 |
| 13 | 23-Mar-14 | 29-Mar-14 |
| 14 | 30-Mar-14 | 05-Apr-14 |
| 15 | 06-Apr-14 | 12-Apr-14 |
| 16 | 13-Apr-14 | 19-Apr-14 |
| 17 | 20-Apr-14 | 26-Apr-14 |
| 18 | 27-Apr-14 | 03-May-14 |
| 19 | 04-May-14 | 10-May-14 |
| 20 | 11-May-14 | 17-May-14 |
| 21 | 18-May-14 | 24-May-14 |
| 22 | 25-May-14 | 31-May-14 |
| 23 | 01-Jun-14 | 07-Jun-14 |
| 24 | 08-Jun-14 | 14-Jun-14 |
| 25 | 15-Jun-14 | 21-Jun-14 |
| 26 | 22-Jun-14 | 28-Jun-14 |
| 27 | 29-Jun-14 | 05-Jul-14 |
| 28 | 06-Jul-14 | 12-Jul-14 |
| 29 | 13-Jul-14 | 19-Jul-14 |
| 30 | 20-Jul-14 | 26-Jul-14 |
| 31 | 27-Jul-14 | 02-Aug-14 |
| 32 | 03-Aug-14 | 09-Aug-14 |
| 33 | 10-Aug-14 | 16-Aug-14 |
| 34 | 17-Aug-14 | 23-Aug-14 |
| 35 | 24-Aug-14 | 30-Aug-14 |
| 36 | 31-Aug-14 | 06-Sep-14 |
| 37 | 07-Sep-14 | 13-Sep-14 |
| 38 | 14-Sep-14 | 20-Sep-14 |
| 39 | 21-Sep-14 | 27-Sep-14 |
| 40 | 28-Sep-14 | 04-Oct-14 |
| 41 | 05-Oct-14 | 11-Oct-14 |
|  |  |  |
